# Cancer stem cells in meningiomas: novel insights and therapeutic implications

**DOI:** 10.1007/s12094-024-03728-6

**Published:** 2024-09-24

**Authors:** Wireko Andrew Awuah, Adam Ben-Jaafar, Simran Karkhanis, Princess Afia Nkrumah-Boateng, Jonathan Sing Huk Kong, Krishitha Meenu Mannan, Vallabh Shet, Shahzeb Imran, Matan Bone, Allswell Naa Adjeley Boye, Sruthi Ranganathan, Muhammad Hamza Shah, Toufik Abdul-Rahman, Oday Atallah

**Affiliations:** 1 Faculty of Medicine, Sumy State University, Sumy, 40007 Ukraine; 2 School of Medicine, University College Dublin, Belfield, Dublin 4, Ireland; 3 School of Medicine, Queen’s University Belfast, Belfast, UK; 4 University of Ghana Medical School, Accra, Ghana; 5 School of Medicine, College of Medical & Veterinary Life Sciences, University of Glasgow, Glasgow, UK; 6 School of Medicine, Queen’s University Belfast, Dentistry & Biomedical Sciences, Belfast, UK; 7 University of Connecticut New Britain Program, New Britain, Connecticut, USA; 8 Salford Royal Hospital, Northern Care Alliance NHS Foundation Trust, Salford, UK; 9 Department of Medicine, University of Cambridge, Cambridge, UK; 10 Department of Neurosurgery, Hannover Medical School, Carl-Neuberg-Strasse 1, 30625 Hannover, Germany

**Keywords:** Meningioma stem cells, Meningioma, Neuro-oncology, Neuro-genetics

## Abstract

Meningiomas (MGs), which arise from meningothelial cells of the dura mater, represent a significant proportion of primary tumours of the central nervous system (CNS). Despite advances in treatment, the management of malignant meningioma (MMG) remains challenging due to diagnostic, surgical, and resection limitations. Cancer stem cells (CSCs), a subpopulation within tumours capable of self-renewal and differentiation, are highlighted as key markers of tumour growth, metastasis, and treatment resistance. Identifying additional CSC-related markers enhances the precision of malignancy evaluations, enabling advancements in personalised medicine. The review discusses key CSC biomarkers that are associated with high levels of expression, aggressive tumour behaviour, and poor outcomes. Recent molecular research has identified CSC-related biomarkers, including Oct-4, Sox2, NANOG, and CD133, which help maintain cellular renewal, proliferation, and drug resistance in MGs. This study highlights new therapeutic strategies that could improve patient prognosis with more durable tumour regression. The use of combination therapies, such as hydroxyurea alongside diltiazem, suggests more efficient and effective MG management compared to monotherapy. Signalling pathways such as NOTCH and hedgehog also offer additional avenues for therapeutic development. CRISPR/Cas9 technology has also been employed to create meningioma models, uncovering pathways related to cell growth and proliferation. Since the efficacy of traditional therapies is limited in most cases due to resistance mechanisms in CSCs, further studies on the biology of CSCs are warranted to develop therapeutic interventions that are likely to be effective in MG. Consequently, improved diagnostic approaches may lead to personalised treatment plans tailored to the specific needs of each patient.

## Background

Meningioma (MG), benign or malignant, is a central nervous system (CNS) tumour commonly found in the meninges of the brain and spinal cord [1]. MGs constitute approximately one-third of all primary CNS tumours in adults [2]. Originating from the meningothelial or arachnoid cap cells of the dura mater, these tumours are frequently observed at the cranial vault, skull base, tentorium cerebelli, and falx cerebri [2]. Malignant meningiomas (MMGs), a rare subset (WHO grade 3), account for about 1.7% of all MGs [1].

Despite significant advances in MG treatment, challenges remain. Conventional magnetic resonance imaging (MRI) is essential for diagnosing and monitoring MMGs but has limitations in accurately determining tumour grade or growth potential [3]. Surgery and radiotherapy are standard treatments, with resection often performed to alleviate symptoms such as seizures and headaches. However, surgery can result in long-term neurological and functional deficits, reducing patients’ quality of life [3, 4]. In addition, complete removal of certain MGs is often hindered by their location and size, posing risks to critical brain regions and blood vessels [3]

Chemotherapy has shown promise in treating MGs, with advances involving drugs such as tyrosine kinase inhibitors, alkylating agents, endocrine drugs, and interferon-targeted molecular pathway inhibitors. However, the detailed functionality and efficacy of these drugs remain underexplored [5]. Addressing these treatment challenges, recent molecular research has introduced novel techniques, such as CSC therapies, aimed at improving outcomes and prognosis.

CSCs are a subset of cancer cells within the tumour microenvironment (TME) that can self-renew and differentiate into various tumour cell types, facilitating metastasis. In CNS tumours, particularly MGs, CSCs are critical markers of tumour growth and metastasis [6]. For instance, the protein CD133/Prominin-1, expressed in haematopoietic stem cells, neural progenitor cells, and ependymal cells in the adult brain, is widely recognised as a CSC biomarker in various cancers, including those of the CNS, lung, and colon [7–9]. Recently, there has been growing evidence of the involvement of CSCs in MGs. In these tumours, high expression of Prominin-1 (also known as CD133) has been associated with rapid cell growth and increased resistance to treatment [9]. CSCs can serve as diagnostic markers for MMGs, aiding in treatment monitoring and prognosis prediction [10]. There is currently a lack of studies focussing specifically on CSCs in MMGs. This gap in research makes it difficult to draw definitive conclusions about whether targeting CSCs in MG could be a viable therapeutic strategy.

Therefore, this review aims to provide a comprehensive analysis of current advances in CSC research and their relevance to MGs, particularly malignant ones, and their potential to revolutionise treatment options for MMGs. By identifying gaps in existing knowledge and research, this review serves as a valuable resource for researchers, clinicians, and medical professionals interested in this field.

## Methodology

This narrative review aims to provide a comprehensive framework of the role of cancer stem cells in meningiomas. Specific inclusion and exclusion criteria were used to ensure a rigorous and comprehensive approach. The inclusion criteria consisted of full text articles written in English. Several databases were used, including PubMed/Medline, EMBASE, the Cochrane Library, and Scopus. Keywords such as ‘cancer stem cells’, ‘meningioma stem cells’, ‘CSCs’, and ‘MgSCs’ were used for a comprehensive database search. References cited in recent reviews on similar topics were also manually reviewed to identify additional sources that could contribute to the search strategy. Standalone abstracts, conference proceedings, case reports, and posters were excluded, with priority given to the inclusion of high-quality and reliable evidence. In addition, the review did not limit the number of studies to provide a comprehensive understanding. It included descriptive, animal model, cohort, and observational studies from both preclinical and clinical settings to provide a holistic perspective. A summary of the methodology used is shown in Table 1.

**Table 1:** Summary of methodology

Methodology steps	Description
Literature search	PubMed/MEDLINE, EMBASE, Scopus and the Cochrane Library
Inclusion criteria	Various study designs including experimental studies, randomised controlled trials, prospective and retrospective cohort studies Studies involving both paediatric and adult populations Studies providing raw data Full-text articles published in English
Exclusion criteria	Non-English studies, stand-alone abstracts, conference proceedings, editorials, commentaries, and letters
Search terms	Key words such as ‘cancer stem cells’, ‘CSCs’, and ‘meningioma stem cells’ and ‘MgSC’ were used for a comprehensive database search
Additional search	A manual search was performed to include references from recently published procedure-specific and disease-specific reviews
Sample size requirement	No strict sample size requirement

## Overview of meningiomas

### Incidence and recent classifications

MGs are the most common benign tumours of the CNS, accounting for about 37.6% of cases worldwide [1]. These tumours represent approximately 50% of all brain tumours. In 2020, the global incidence of MGs was 3.5 per 100,000 population, with a higher incidence in women, particularly middle-aged women [11]. The incidence increases with age, with younger adults and children accounting for 0.4–4.6% of cases. MGs are more prevalent in the white population, with a rate of approximately 9.52 per 100,000 people, compared to 3.34 per 100,000 in the African population. Asians and Hispanics have intermediate rates of 5.43 and 7.02 per 100,000 people, respectively [12]. In addition, African Americans have a higher risk of tumour recurrence [13].

**Fig. 1:**
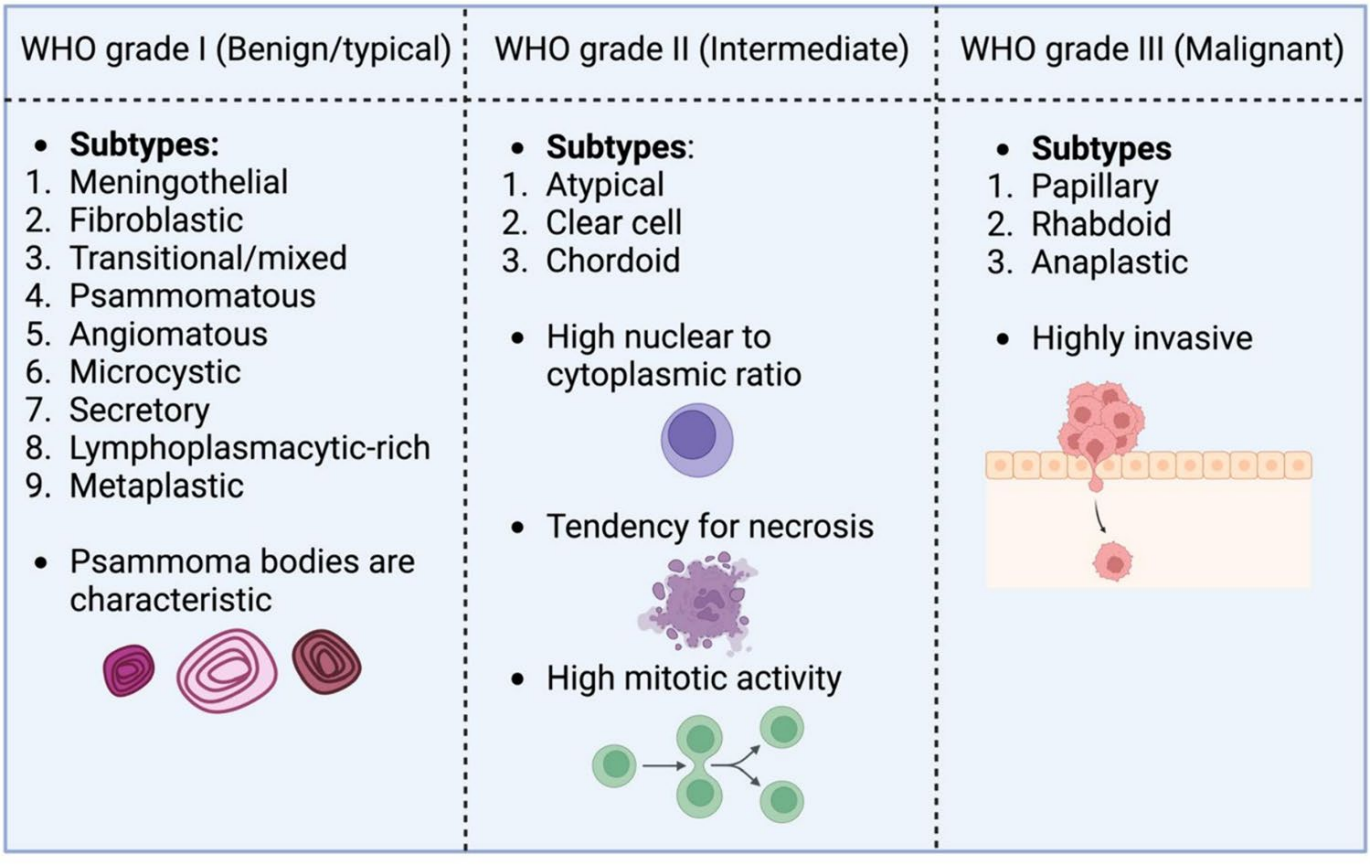
An overview of the World Health Organisation grades of meningiomas (Created with Biorender.com).*WHO*: World Health Organisation

According to the latest WHO classification, MGs are divided into three grades based on their growth rate and likelihood of recurrence [3]. These classifications include WHO grade 1 (benign or typical, accounting for approximately 80–81%), WHO grade 2 (atypical, 17–18%), and WHO grade 3 (malignant, 1.7%) [1]. MMGs have higher recurrence rates than benign and atypical MGs, with rates of 50–94% for grade 3, compared to 7–25% and 29–52% for benign and atypical MGs, respectively [1]. There are 15 histological subtypes within these 3 groups, differentiated by cell type. Grade 1 includes 9 variants, such as meningothelial, fibroblastic, transitional or mixed, psammomatous, angiomatous, microcystic, secretory, lymphoplasmacytic-rich, and metaplastic subtypes. A characteristic feature of these tumours is the formation of psammoma bodies after mineralisation from meningothelial cells, which can lead to excessive bone growth and exostosis of the adjacent bone [1]. Grade 2 MGs include atypical, clear cell, and choroid variations, which are intermediate-grade lesions with a high nuclear-to-cytoplasmic ratio, large nuclear size due to increased chromatin, a tendency for necrosis, and high mitotic activity. Grade 3 malignant lesions are divided into papillary, rhabdoid and anaplastic histological subtypes. These subtypes are characterised by a high rate of invasion into surrounding tissues, similar to high-grade carcinomas, sarcomas, craniopharyngiomas, lymphomas, and ependymomas [1, 13]. The WHO grading of MGs with its subtypes has been summarised in Fig. 1.

### Genetics and pathogenesis

MGs develop from the meningeal layers of the CNS; specifically, the arachnoid barrier cells are located within the leptomeninges, with approximately, 90% found above the tentorium and the remaining 10% distributed between the areas below the tentorium and along the spinal cord [1, 3, 14]. Chromosomal mutations play a significant role in the development of MGs, which can involve single or multiple deletions, with the latter leading to greater metastasis and faster tumour growth. Some MGs are sporadic, while others are inherited and linked to genetic abnormalities. The most common genetic factor in benign MGs is mutations in the *neurofibromatosis* (*NF2*) gene. Cytogenetic and molecular analyses reveal that the loss of chromosome 22, where the *NF2* gene is located at 22q12.2, is a primary event in the early development of these tumours [15]. Other genetic drivers include Krüppel-like factor 4 (KLF4), tumour necrosis factor receptor-associated factor 7 (TRAF7), smoothened frizzled class receptor (SMO), and v-Akt murine thymoma viral oncogene homolog 1 (AKT1) [16, 17]. The loss of chromosome 22q is the most frequent cryptogenic reconfiguration, occurring in 60–70% of all MG cases. In addition, losses of chromosomes 1p, 9p, and 14q are observed in advanced cases of MGs [18].

Syndromes associated with a higher risk of developing MGs include von Hippel–Lindau syndrome (VHL) and multiple endocrine neoplasia type 1 (MEN 1) [1, 19]. Individuals with these genetic mutations are more likely to develop multiple or MMGs, which can also occur in young children [19]. Malignant progression follows the theory of clonal evolution, involving a series of chromosomal gains and losses leading to subclones with increasing growth advantage [20–22]. Anaplastic MGs exhibit complex genetic changes with chromosomal losses on 1p, 6q, 10q, 14q, and 18q. Epigenetic modifications occur with higher levels of CpG island hypermethylation, which are associated with the progression of MMG [18, 23].

**Fig. 2:**
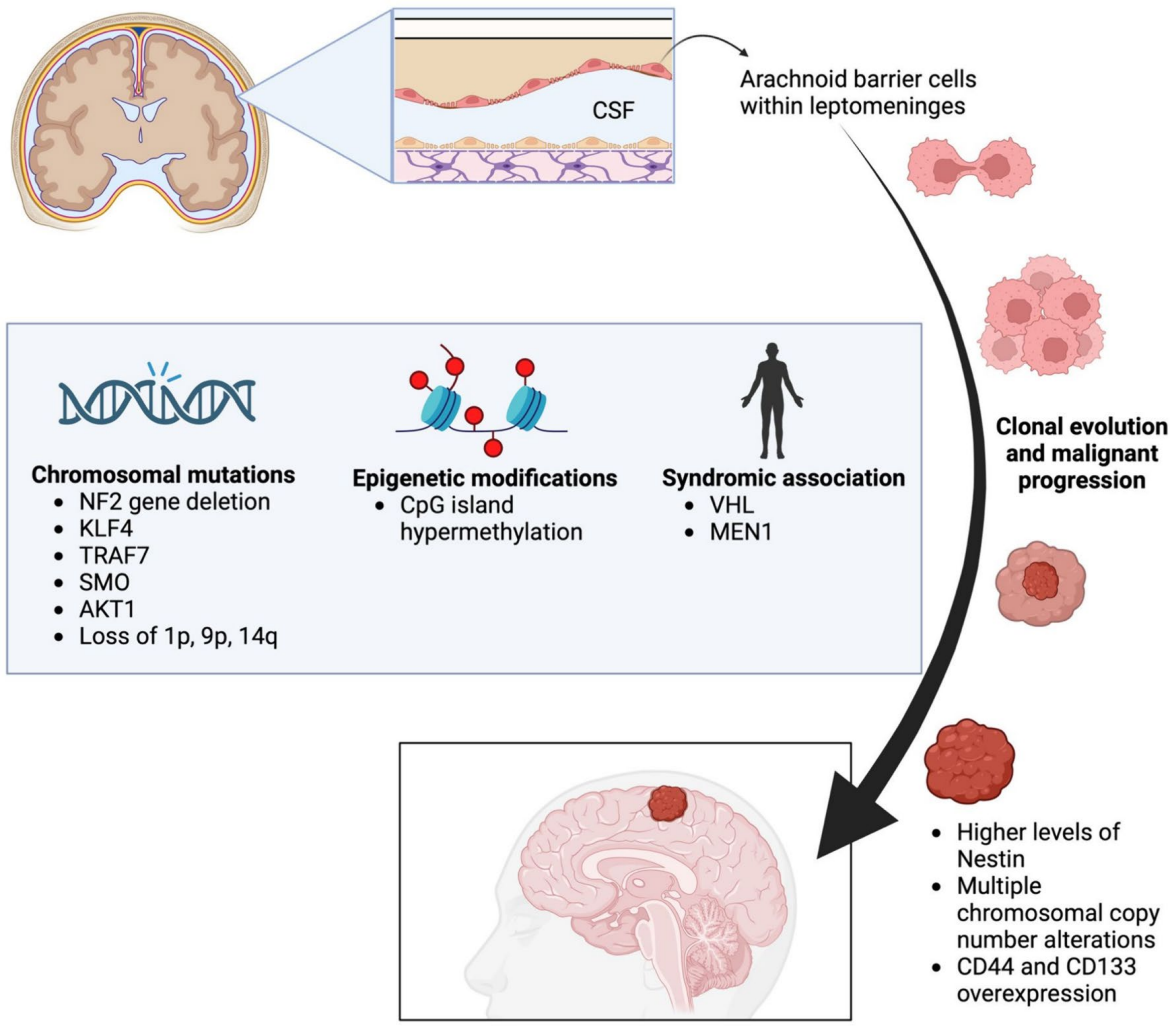
A comprehensive review of the genetics and pathogenesis of meningiomas (Created with Biorender.com).*CSF*, cerebrospinal fluid; *NF2*, neurofibromatosis 2; *KLF4*, Krüppel-like factor 4; *TRAF7*, tumour necrosis factor receptor-associated factor 7; *SMO*, smoothened; *AKT1*, v-Akt murine thymoma viral oncogene homolog 1; *VHL*, Von Hippel–Lindau; *MEN1*, multiple endocrine neoplasia type 1

Furthermore, higher levels of Nestin, a type IV intermediate filament, have been discovered in higher grade MGs (grades II and III) [24]. MMGs tend to have multiple chromosomal copy number alterations, consistent with the accumulation of mutations [19]. Research indicates that CD44 overexpression in MG cells is associated with increased invasiveness and anaplasia [25]. In addition, a positive relationship has been found between CD133 overexpression and invasion, suggesting an important role for CD133 in MGs. This indicates that CD133-positive cells may contain more CSCs [25]. Figure 2 illustrates the genetics and pathogenesis of MGs.

## Overview of cancer stem cells

### History and evolution

The concept of CSCs has significantly evolved since its inception, mirroring advances in understanding cancer’s complexities. In the early nineteenth century, Julius Cohnhein observed the resemblance between teratocarcinomas and embryonic tissues. He then put forward the *“embryonal rest” theory*, suggesting that cancer could originate from embryonic cells that remain in the adult organism [26]. This laid the groundwork for the *stem cell theory* of cancer. Throughout the nineteenth century, theories and observations linked tumours to embryonic tissue growth. In the 1960s, Barry Pierce’s work on mice provided substantial evidence for the cancer stem cell concept by showing that certain cancer cells could induce tumours that resembled the tissues from which they originated [27].

The modern era of CSC research began in the late nineteenth century with a breakthrough study on acute myeloid leukaemia (AML). John Dick and colleagues identified that a subset of CD34 + and CD38- cells from AML patients could initiate leukaemia in diabetic mice, highlighting the critical role of CSCs in cancer progression and therapy resistance [28]. Further research has led to a better understanding of CSCs and their identification in various types of cancers, including solid tumours. This was first demonstrated in human breast cancer and has been replicated in brain, colon, and pancreatic cancer [29–32].

The current CSC theory posits that a small subpopulation of cells within a tumour has the capacity to self-renew and differentiate [33]. Recent work into understanding the complex biology of CSC aims to develop targeted therapies to effectively eradicate these cells. One major breakthrough has been the identification of common signalling pathways between cancer and stem cells—such as the JAK/STAT, MAP-kinase/ErK, NOTCH, NF-κB, P13K/Akt, TGF-β, and Wnt pathway [34]. Moreover, there has been an increased interest in understanding the role of CSCs in initiating cancer, apoptosis, recurrent and their metastatic capacity. One major focus has been the detection of specific surface markers such as CD133, CD44 and Oct-4, which could lead to more precise and effective treatments [35, 36].

### CSCs origin

At present, there is still controversy about the mechanisms involved in the formation of CSCs. One hypothesis is that CSCs can arise from differentiated cells through a process of dedifferentiation. Differentiated cells have the ability to revert to a stem-like state under certain conditions, thereby acquiring the ability to self-renew [8, 37]. This process is similar to the reprogramming of somatic cells into induced pluripotent stem cells (iPSCs), where differentiated cells regain their pluripotency. In cancer, this process is reversed by oncogenic signals, leading to the generation of CSCs that contribute to tumour heterogeneity and resilience [37]. Another hypothesis is that CSCs are derived from adult tissue-resident stem cells. These stem cells have the ability to self-renew, making them targets for transformation into CSCs following oncogenic mutations. Since these cells already have stem-like properties, they can give rise to CSCs when they acquire additional mutations. This leads to the sustained growth and spread of tumours, supporting the theory that tumour organisation is initiated by a small population of SC-like cells at the top, giving rise to diverse cell types within the tumour [8].

The process of dedifferentiation, in which more differentiated cancer cells revert to a stem-like state, is another key hypothesis for the origin of CSCs. Dedifferentiation is often triggered by factors such as hypoxia, inflammation and interactions with stromal cells. This allows non-stem cancer cells to acquire stem cell properties, including self-renewal and the ability to direct tumour growth. This process of tumour cell plasticity highlights the ability of cancer cells to switch between different states [37]. However, a more recent hypothesis involves the role of cellular energy depletion in the formation of CSCs. This model suggests that a reduction in ATP levels can trigger DNA instability, leading to the dedifferentiation of cancer cells into a stem-like state [38]. It is thought that the energy crisis within cells disrupts normal metabolic processes, causing them to revert to more resilient forms that can proliferate under more extreme conditions [38].

### CSCs heterogeneity and plasticity

Cancer manifests in multiple subtypes, each possessing distinct characteristics and biological significance. Tumour cells exhibit variability in growth rates, cell surface marker expression, genomic profiles, and resistance to therapy. These diverse cell types adapt to and interact with the microenvironment to ensure survival and proliferation [39]. Tumour behaviour and treatment outcomes are profoundly influenced by this heterogeneity. CSCs significantly contribute to tumour heterogeneity due to their capacity for self-renewal and differentiation into hierarchical cell types, ranging from highly tumorigenic cells to intermediate and terminally differentiated progenitors [39]. This variation can occur within a single tumour (intratumoral) or between different tumours (intertumoral) [40].

Intratumoral heterogeneity aids in distinguishing cancer types based on histology, genetic makeup, and specific markers, which is crucial for determining clinical prognosis [39]. In contrast, intratumoural heterogeneity introduces uncertainties in prognosis and treatment outcomes, making it a significant contributor to cancer progression and poor treatment results [41]. This paradigm of tumour heterogeneity is further complicated by the ability of CSCs to reversibly transition between non-cancer stem cells to cancerous stem cells [42]. This inter-transition, named plasticity, of CSC is regulated by extrinsic factors like complex signalling pathways in the TME and intrinsic factors such as genetic and epigenetic modifications.

Interactions with the TME drive the phenotypic plasticity of cancer cells through specific chemical signals and cellular crosstalk. For instance, NOTCH1 signalling modulates plasticity in mesenchymal stem cell-derived fibroblasts, thereby impacting melanoma aggressiveness [43]. Kim et al. (2018) used flow cytometry to demonstrate how hypoxia in the TME can determine the fate of breast CSCs [44]. The study found that the hypoxic TME in breast cancer enhances CSC characteristics through the activation of the PI3K/AKT pathway [44]. These CSC traits are stable and persist even after re-implantation, highlighting that hypoxia in vivo is crucial for encouraging malignant progression and therapy resistance [44].

Epigenetic modifications alter gene expression and contribute to cellular plasticity. These modifications include alterations in histones, DNA methylation pattern and chromosomal rearrangements [45]. Chromosomal modifications like ZEB1, a transcription factor, acts as a switch that enables human breast cancer cells to transition from non-CSC to CSC states. Furthermore, chromatin structure is also altered by histone acetylation, regulated by histone acetylases (HATs). Proteins involved in DNA methylation, known as methyltransferases, add methyl groups to cytosine residues of DNA, typically at CpG dinucleotides. These proteins play a crucial role in escaping immune detection and activating signalling pathways [46]. In addition, they are recognised as crucial drivers in the formation of CSCs [43]. For instance, mutations in DNMT3A (a DNA methyltransferase) have been linked to the development of acute myeloid leukaemia (AML), highlighting the role of DNA methylation in cellular plasticity [47].

CSCs are key drivers of tumour progression and therapy resistance due to their heterogeneity and plasticity. Through the creation of an immunosuppressive environment, they are able to avoid immune surveillance by expressing immune checkpoint proteins and recruiting suppressive immune cells [48]. The TME increases the radioresistance of CSCs and supports their survival, particularly under hypoxic conditions. This in turn contributes to treatment failure and relapse in MG patients. In addition, mitochondria play a critical role in CSC biology, with high mitochondrial content enabling these cells to maintain energy production, resist apoptosis and withstand oxidative stress [49]. Understanding these mechanisms is essential for the development of targeted therapies to effectively combat CSCs.

Recent research on the survival and treatment resistance of CSCs highlights the critical role of mitochondrial dynamics, focussing on mitophagy and mitochondrial biogenesis. To clear damaged mitochondria, CSCs utilise mitophagy to maintain cellular homeostasis and avoid apoptosis [50]. Particularly under hypoxic conditions, this adaptation allows CSCs to shift their metabolism from oxidative phosphorylation (OXPHOS) to glycolysis, which aids their survival and resistance to conventional therapies [51]. Mitochondrial biogenesis further supports CSCs by replenishing their mitochondrial pool to meet the high energy demands required for their continued growth and proliferation. The coordinated regulation of these processes not only contributes to the resilience of CSCs, but also poses a significant challenge in the development of effective treatments aimed at their eradication [50, 51].

### Therapeutic resistance of CSCs

The plasticity of CSCs is characterised by their ability to dynamically adapt to various microenvironmental conditions and undergo epigenetic modifications. This plasticity, combined with cell heterogeneity, has endowed CSCs with resilience, allowing them to resist even the most advanced therapies, thereby contributing to ongoing challenges in cancer treatment [52]. Phenotypic CSCs have demonstrated enhanced resistance to chemoradiotherapy.

#### Resistance to radiotherapy

CSCs have shown significant resistance to radiotherapy, which is a common treatment for various cancers. Bao et al., (2006) observed that CD133 + glioblastoma (GB) cells exhibited significantly greater resistance to ionising radiation compared to their CD133 − counterparts [53]. Moreover, CSCs also possess efficient DNA repair mechanisms that render them excellent DNA protection from therapeutic drugs [54].

#### Resistance to chemotherapy

CSCs also exhibit remarkable resistance to chemotherapeutic agents. In the same study by Liu et al. (2006), CD133 + GB cells were found to be significantly more resistant to chemotherapeutic drugs such as temozolomide, carboplatin, paclitaxel, and etoposide than their CD133 − counterparts [55]. This resistance of CSCs is owed to multidrug resistance (MDR) transporters such as adenosine triphosphate-binding cassette (ABC) pumps that facilitate efflux of chemotherapeutic drugs, reducing their intracellular concentration and effectiveness [56].

**Fig. 3:**
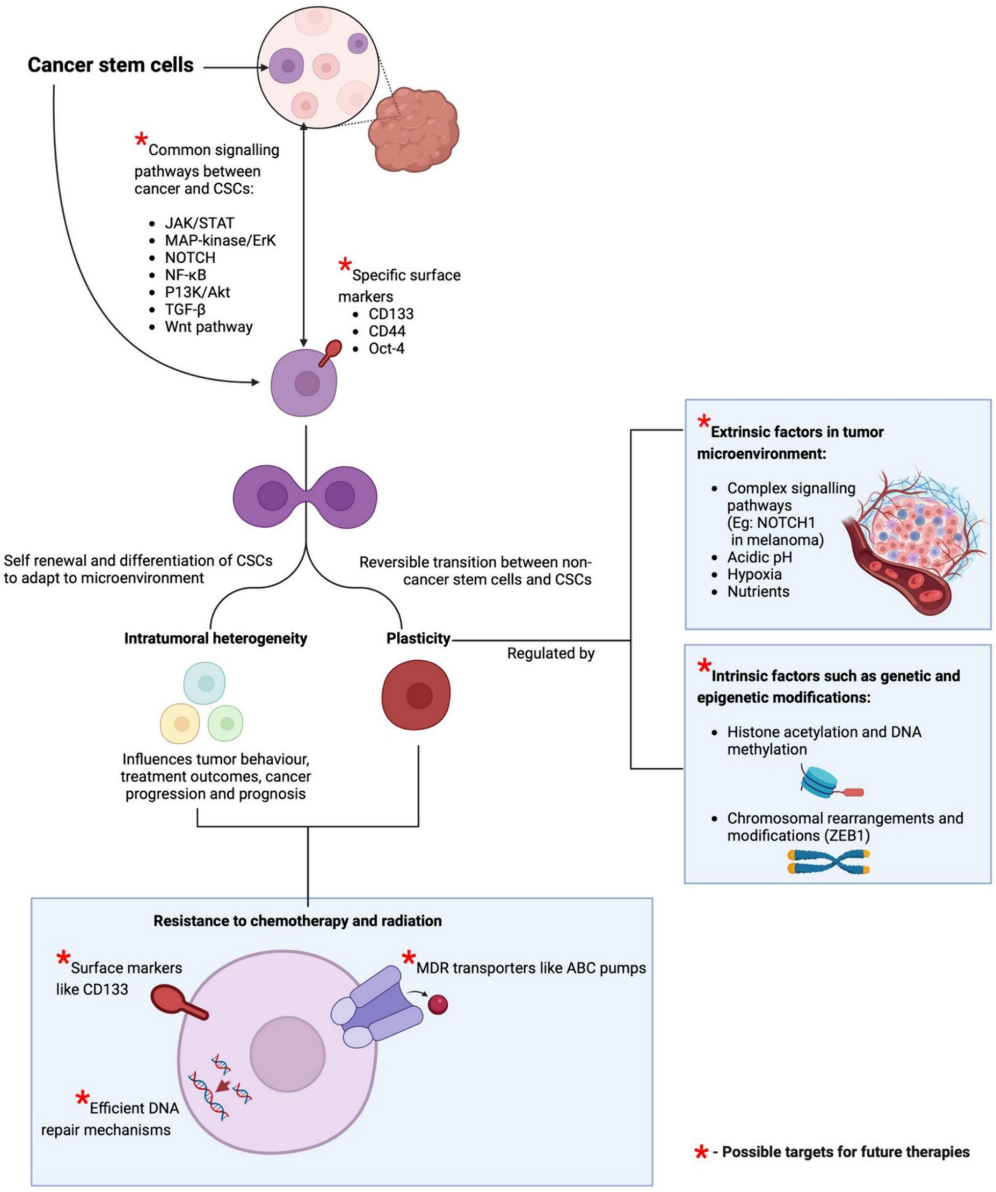
Overview of Cancer Stem Cell characteristics and potential drug targets (Created with Biorender.com).*CSCs*: cancer stem cells, *DNA*: deoxyribonucleic acid, *ABC*: adenosine triphosphate-binding cassette, *MDR*: multidrug resistance, *JAK*: Janus kinase, *STAT*: signal transducer and activator of transcription, *MAP-kinase*: mitogen-activated protein kinase, *NOTCH*: neurogenic locus notch homolog protein, *ERK*: extracellular-signal-regulated kinase, *PI3K*: phosphatidylinositol-3 kinase, *TGF-β*: transforming growth factor beta, *ZEB1*: zinc finger E-box binding homeobox-1, *NF-kB*: nuclear factor-kappa B, *WNT*: wingless-related integration site

Moreover, CSCs remain in a quiescent state, rendering them less susceptible to chemotherapy and radiotherapy-induced DNA damage [57]. Future research should focus on the development of drugs that specifically target these pathways and stem characteristics. Figure 3 provides a detailed illustration of CSC characteristics and various pathways that could serve as potential targets for future therapies.

## *Cancer* stem cells and meningioma

### Clinical importance of CSCs in MGs

Recent studies have highlighted the role of CSCs in the progression and treatment resistance of meningiomas. Shivapathasundram et al. (2018) reviewed the growing evidence proposing that MGs contain a distinct population of CSCs that contribute to the aggressive clinical behaviour of these tumours [58]. These cells are characterised by specific markers, including CD133, which are associated with higher tumour grades and poorer outcomes. CSCs are not only involved in the initial formation of the tumour, but also play a significant role in its ability to evade standard treatments such as surgery and radiotherapy [58]. Further studies have isolated and characterised stem-like cells from human MGs, revealing their significant resistance to conventional therapies such as chemotherapy and radiotherapy. These MgSCs showed higher tumourigenicity in in-vivo models and exhibited markers, such as CD133, that are critical for maintaining their stem-like properties and resistance to treatment [59].

In addition, different CSC subpopulations have been shown to contribute to tumour behaviour and response to therapy. Sudanese meningioma patients have shown inconsistent expression of stem cell markers such as CD44, CD73 and CD105 in different meningioma samples [60]. In support of this, Barbieri et al. (2023) showed that CSCs within MGs exhibit different levels of responsiveness to the CXCL12-CXCR4/CXCR7 chemokine axis, which is known to regulate tumour invasiveness and recurrence. Their study found that CSCs with higher responsiveness to this chemokine axis were more likely to contribute to aggressive tumour behaviour, suggesting that targeting these specific CSC subpopulations may be key to developing more effective treatments for high-risk meningiomas [61].

### Crosstalk between meningioma stem cells and their niche

#### Cellular components

CSCs are known to actively communicate with their surrounding TME, a phenomenon that is particularly evident in MGs. The TME, composed of diverse cellular components such as endothelial cells, pericytes, myeloid cells, mesenchymal stem cells, immune cells and fibroblasts, plays a critical role in regulating CSCs [62]. Within this environment, stromal cells support the stemness and survival of CSCs by secreting growth factors, interleukin-6, adhesion molecules and cytokines. These secreted factors enhance the properties of CSCs, including self-renewal, proliferation and resistance to therapy, which in turn leads to increased metastasis. In addition, MgSC crosstalks with immune cells through the secretion of cytokines and chemokines [63]. A study by Barbieri et al. (2023) showed that pharmacological blockade of CXCR4 and CXCR7 selectively impaired CSC-related functions within the MgSC population [61]. The CXCL12-CXCR4 and CXCL11/12-CXCR7 pathways are significantly upregulated in several tumour CSCs, including those in MGs [64]. In MGs, specific cell subpopulations with CSC-like properties rely on the CXCL12/CXCL11/CXCR4/CXCR7 axis to drive aggressive behaviours such as increased cell proliferation, invasiveness and neovascularisation [61]. These findings suggest that chemokine-producing immune cells may have a direct impact on the stem-like properties of human MG cells, although the precise mechanisms of this crosstalk remain unclear.

In addition to their self-renewal capacity, MgSCs express markers typically found in embryonic stem cells. Analysis of data from 11 patients with WHO grade 1 MG revealed that embryonic stem cell markers were present in the endothelial cell and pericyte layers in all samples [58]. Endothelial cells are critical for the development of tumour angiogenesis. In addition, the study showed that the expression pattern of the renin–angiotensin system (RAS), including the pro-renin receptor (PRR), angiotensin-converting enzyme (ACE), angiotensin II receptor 1 (ATIIR1) and angiotensin II receptor 2 (ATIIR2), on the microvessels mirrored that of embryonic stem cells. The RAS, an endocrine system, regulates several physiological processes, including blood pressure and electrolyte balance [65]. Its components play a role in cell proliferation, angiogenesis and apoptosis. In particular, ATIIR1 has been implicated in tumourigenesis through the vascular endothelial growth factor (VEGF) signalling pathway and mitogen-activated protein kinase phosphorylation, which are critical for tumour progression [66, 67]. Although the exact mechanism of this interaction is not fully understood, these findings suggest a potential crosstalk between MgSCs and endothelial cells that may contribute to the proliferation, survival and progression of MGs.

#### Extracellular matrix

In addition, the extracellular matrix (ECM) within the MG TME plays a critical role in supporting intratumoral signalling and trafficking, thereby enhancing CSC function, particularly in solid tumours [62]. MG cells produce several ECM proteins, including laminin, tenascin, fibronectin, collagens, galectin-3, and matrix metalloproteinases MMP-2 and MMP-9 [68, 69]. These ECM proteins are closely associated with the increased stemness of cancer cells, promoting their survival, proliferation and aggressiveness by providing essential physical and mechanical cues [70]. The interaction between CSCs and their microenvironment is highly dynamic, with CSCs secreting factors that influence stromal and immune cells, establishing a feedback loop that sustains tumour growth and resistance [62]. Another proposed pathway explaining the stemness involves hypoxia, where tumour cells adapt to low oxygen conditions by activating the transcription factor hypoxia-inducible factor (HIF-1α), altering cell metabolism. This adaptation, along with ECM remodelling, leads to changes in ECM stiffness and topography, further contributing to invasion and metastasis [71]. This suggests that MgSCs may crosstalk with the ECM, potentially enhancing their own survival and promoting MG growth within the TME.

### Meningioma stem cells’ potential role in resistance to treatment, recurrences and metastases

MGs have been shown to contain CSCs, which are highly resilient cancer cells that utilise deregulated stem cell expression profiles and contribute to tumour recurrence [58, 72]. CSCs are associated with recurrence due to their ability to resist treatment and promote metastasis [73].

#### Chemotherapy resistance

In MGs, stem cell-like cells may contribute to treatment resistance, including chemoresistance [59]. MgSCs can induce resistance through several mechanisms, including epithelial-to-mesenchymal transition (EMT), which activates neural stem cell (NSC) signalling pathways and induces MgSC characteristics such as entering a quiescent state [74]. Quiescent cells are not affected by most conventional treatments, which typically target actively dividing cells [75]. In addition, CSCs often express high levels of ATP-binding cassette (ABC) transporters, which pump toxic substances out of the cell [76, 77]. CSCs also escape apoptosis by mutating or inactivating genes that regulate the cell cycle and apoptosis and possess highly active DNA damage response systems [53, 78, 79]. Although definitive studies are lacking, these findings provide important insights into potential mechanisms by which MgSCs might promote treatment resistance. These stem cell-like cells have been found to be more resistant to vincristine than normal cells at various doses, a phenomenon commonly observed in WHO grade II and III MGs [59, 80].

#### Radiotherapy resistance

The role of MgSCs in treatment resistance extends beyond chemotherapy to include radiotherapy. Research suggests that factors released by both CSCs and the TME contribute to radioresistance [81]. In addition, CSCs have been shown to inhibit cell cycle progression by arresting cells in the G0 phase, further enhancing radiation resistance [82]. Although the specific involvement of MgSCs in radioresistance in MGs has not been conclusively demonstrated, the established association between CSCs and radioresistance suggests that MgSCs may also play a role in this phenomenon.

#### Genetic factors and recurrence

Genetic factors play an important role in the recurrence of MGs. For example, methylation of the promoter region of the *GSTPE* gene has been associated with a higher risk of recurrence [80]. In addition, although the protein p300 has not been specifically associated with MgSCs in MGs, its overexpression in certain MG cells suggests that it may serve as a potential biomarker for tumour recurrence [83]. In addition, elevated levels of stem cell-related proteins such as NANOG, Oct-4 and Sox2 have been implicated in increased tumour aggressiveness and metastasis [84]. These proteins, together with dysregulated expression of genetic factors, are thought to be key drivers in the pathogenesis, recurrence and metastasis of MGs.

**Fig. 4:**
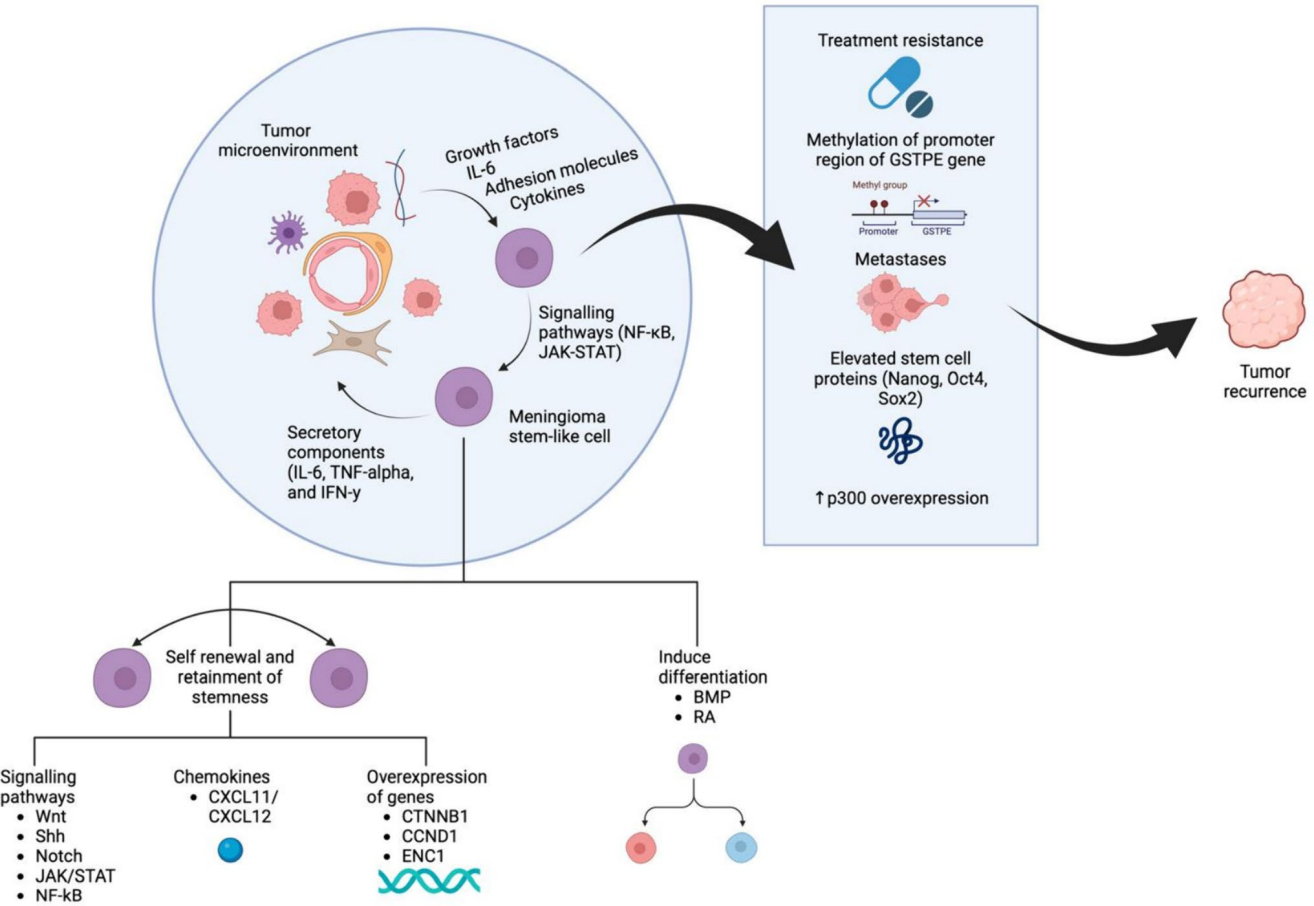
Correlation between cancer stem cell renewal, resistance, and recurrence (created with Biorender.com). *IL-6*: interleukin-6, *Nf-kB*: nuclear factor-kappa B, *JAK*: Janus kinase, *STAT*: signal transducer and activator of transcription, *TNF-alpha*: tumour necrosis factor-alpha, *WNT*: wingless-related integration site, *SHH*: sonic hedgehog, *NOTCH*: neurogenic locus notch homolog protein, *IFN-γ*: interferon gamma, *CXCL*: CXC chemokine ligand, *CTNNB*: catenin beta, *CCND*: Cyclin D, *ENC*: ectodermal-neural cortex 1, *BMP*: bone morphogenic protein, *RA*: retinoic acid, *GSTPE*: glutathione s-transferase P, *Oct-4*: octamer-binding transcription factor 4, *Sox2*: sex-determining region Y-box 2

Furthermore, specific CSC markers in MGs such as CD133, Sox2, Nestin and Frizzled 9 have been shown to confer greater resistance to drug treatments such as cisplatin or etoposide [85]. In particular, the expression of CD133 and nestin in grade II/III MGs has been associated with higher recurrence rates [86]. Figure 4 summarises the relationship between the CSC renewal, resistance and recurrence.

### Meningioma stem cell biomarkers

A diverse spectrum of biomarkers has been identified as contributory to the pathology of MgSCs, and identifying these may have a potential pivotal role in pathogenesis and management. These markers have been discussed below.

#### Oct-4

Oct-4 is a homeodomain transcription factor that plays a key role in the self-renewal of undifferentiated stem cells and is a master gene of stem cell pluripotency. Its levels are tightly controlled and even small variations can predict stem cell lineage [87]. Recent studies have shown that Oct-4 can also restore pluripotency in somatic cells, and it has been investigated for its role in the generation of induced pluripotent stem cells [88]. The role of Oct-4 in the pathogenesis of MGs has been extensively studied. Immunohistochemical analysis has shown that co-expression of Oct-4, along with other transcription factors including Sox2 and NANOG, may be a key genetic driver in the initiation of MGs and it has been identified in both low- and high-grade MG samples [58, 72]. Recent studies have demonstrated a strong association between the magnitude of Oct-4 expression and both tumour grade and recurrence, with Oct-4 positivity being significantly higher in high-grade MGs and recurrent cases [89]. While studies support the presence of Oct-4 in CSCs, it does not appear to be exclusive to this population. Recent work has shown that pluripotency transcription factors, including Oct-4 and Sox2, are present in non-stem tumour cells and normal meningeal cells, limiting the potential role of these factors as differentiators between stem and non-stem cells [61].

#### Sox2

The transcription factor Sox2 is essential for maintaining the self-renewal capacity of NSCs and the undifferentiated state of CSCs [90–92]. Sox2 plays a critical role in physiological and pathological processes, including cell proliferation, migration, invasion, tumourigenesis and anti-apoptosis [93]. In cancer, it is crucial for maintaining the pluripotency and stem cell properties of CSCs [94, 95]. In MGs, Sox2 is preferentially expressed in high-grade tumours and correlates with clinical behaviour, with expression being highest in grade 3 tumours [89]. Sox2-expressing stem cells have been observed in atypical MGs and in stem cells cultured from these tumours [96]. In addition to drug resistance, Sox2 expression at diagnosis is strongly associated with an increased risk of surgical recurrence and poor prognosis in MGs [97]. While Sox2 silencing has been shown to stop proliferation in other neurological tumours, further research is needed to confirm these findings in MGs [98].

#### NANOG

NANOG is a homeobox-binding protein found in embryonic stem cells (ESCs) that plays a key role in the transcriptional regulation of self-renewal and pluripotency [72, 99]. It coordinates self-renewal and differentiation of ESCs and is also involved in metastasis and carcinogenesis [72, 99]. Similar to Sox2, NANOG is a transcription factor essential for maintaining pluripotency and stemness. Downregulation of NANOG leads to reduced pluripotency and tumourigenicity [100], whereas its overexpression is significantly associated with poor prognosis in several cancer types [101, 102]. Freitag et al. (2017) found that NANOG expression is almost twice as high in high-grade MGs compared to low-grade ones. Despite its self-regulatory mechanisms, NANOG expression is also modulated by Sox2 and Oct-4 [72]. While this genetic interplay has been less explored in MgSCs, understanding it could provide insights into the pathogenesis and therapeutic implications of NANOG in MG, an area that remains largely unexplored and warrants further investigation.

#### CD133

CD133, also known as Prominin-1, is a five-transmembrane glycoprotein that is critical in several cancer-related processes, including tumorigenesis, metastasis, and resistance to chemotherapy and radiotherapy [103]. It is commonly expressed by stem cells in MGs and is typically found in embryonic NSCs, radial glial cells and ependymal cells in the adult brain [59, 96, 103]. CD133 is thought to interact with specific gangliosides to modulate cell–cell contact in a cell cycle-related manner [104, 105]. CD133, Sox2, Nestin and Frizzled 9 have been shown to confer greater resistance to drug treatments such as cisplatin or etoposide [85]. Although the exact mechanism by which CD133 influences MgSCs to promote resistance remains unclear, its higher expression in MG cell lines is associated with increased cell proliferation and drug resistance [58, 106]. CD133 is the most commonly used marker for stem cell isolation in neurological tumours, and its expression is prevalent in MMGs and correlates with aggressive proliferation and reduced progression-free survival [86, 107].

#### Nestin

Nestin is a class VI intermediate filament protein that was first detected in NSCs during development [108]. In MGs, nestin, a marker for MgSCs, is often associated with more malignant forms of the tumour. It is a key determinant of proliferation rates and is often overexpressed along with other biomarkers to increase malignancy [109, 110]. Galani et al. investigated nestin expression in 17 patients with MGs using qRT-PCR and found that nestin levels were significantly higher in atypical and anaplastic MGs compared to benign cases [24]. Similarly, Xiao et al. reported that while all atypical and anaplastic MGs and the majority of benign MGs were positive for nestin, expression was significantly higher in non-benign MGs [109]. Consistent with the findings for CD133, higher nestin expression in grade II/III MGs is associated with reduced progression-free survival [86]. However, the proportion of Ki67-positive cells, a marker commonly used to assess cell proliferation, that were Nestin-negative was higher in grade II/III MGs, although nestin expression increases with MG grade [9, 24, 111, 112]. Therefore, further research is needed to clarify the relationship between nestin expression and tumour behaviour, including its role in proliferation and progression.

#### c-MYC

Studies have shown that c-Myc expression in MGs varies with tumour grade and recurrence. Low-grade MGs generally test negative for c-Myc, whereas recurrent high-grade lesions often express this oncogene, suggesting its association with tumour progression and recurrence [113, 114]. As a key regulator of cell proliferation and growth, c-Myc is implicated in both normal and neoplastic cells [113]. Its role in stem cell biology further highlights its potential as a link between malignancy and ‘stemness’ [88]. In immunohistochemical studies, c-Myc has been observed in the nuclear or perinuclear regions of atypical and anaplastic MGs. In addition, there is a positive correlation between c-Myc levels and cell proliferation in malignant or recurrent MGs [115–117].

#### CD44

CD44 is a transmembrane glycoprotein commonly associated with various cancers, where it plays a critical role in tumour invasion and metastasis [118]. In MGs, CD44 is known to regulate essential biochemical processes such as tumour cell adhesion, angiogenesis, proliferation and inflammation [119]. Studies have shown a positive correlation between elevated CD44 levels and higher WHO MG grades, highlighting its potential as a marker of tumour severity and a target for therapeutic intervention [120, 121]. Furthermore, research by Dai Kamamoto et al. (2019) showed that CD44 expression is positively associated with areas of high tumour cell density in most cases of high-grade MGs, further highlighting its importance in the progression of these tumours [122]. The types and functions of different MgSC markers are summarised in Table 2.

**Table 2:** Meningioma stem cell markers and their functions

Biomarker	Role in MgSCs
Oct-4 [58, 61, 72, 87–89]	Regulates pluripotency and self-renewal of undifferentiated stem cells and stem cell pluripotency It plays a key role in the initiation of meningiomas and its expression correlates with tumour grade Co-expression with Sox2 and Nanog is essential in the initiation of meningiomas It is present in both high- and low-grade meningiomas
Sox2 [94, 98]	Maintains pluripotency and self-renewal It is found at high levels in high-grade meningiomas, which is associated with poor outcomes
NANOG [72, 100, 101]	A transcription factor that plays a role in maintaining pluripotency Its overexpression is linked to poor prognosis and is notably higher in high-grade meningiomas It is regulated by SOX2 and Oct-4
CD133 [85, 86, 106, 107]	Common stem cell marker in malignant meningiomas It is associated with aggressive behaviour and poor survival Inversely correlated with progression-free survival
Nestin [109, 110]	Prevalent in malignant meningiomas and is associated with increased proliferation Acts as a determinant of the rate of proliferation It is routinely overexpressed with other biomarkers to synergistically stimulate malignancy
c-MYC [88, 113–117]	Has a role in ‘stemness’ of stem cells Frequently expressed in high-grade meningioma Positive correlation between c-Myc levels and cell proliferation in malignant or recurrent meningiomas
CD44 [118–122]	Has a role in tumour invasion and metastasis Regulates tumour cell adhesion, angiogenesis, proliferation and inflammation Studies show positive correlation between CD144 levels and WHO meningioma grades

*MgSCs*, Meningioma stem cells; *Oct-4*, octamer-binding transcription factor 4; *Sox2*, sex-determining region Y (SRY)-box 2; *Nestin*, neuroepithelial stem cell protein; *WHO*, World Health Organisation

## Discussions and prospects on strategies targeting meningioma stem cells

### Therapies targeting MgSC markers and their related signalling pathways

CSCs are characterised by their capacity for self-renewal, differentiation, and significant resistance to conventional cancer therapies. Understanding and disrupting the signalling pathways critical for their maintenance and function is essential. Key pathways such as Wnt, Notch, and HH have been extensively studied for their roles in CSC therapies.

The HH pathway involves the interaction of HH ligands, namely sonic hedgehog (SHH), desert hedgehog, and Indian hedgehog (IHH) with the patched receptor (PTCH1), and dampening smoothened (SMO) protein repression. This leads to the accumulation of Gli transcription factors including, Gli1, Gli2, and Gli3, stimulating target gene expression that mediates cell proliferation and differentiation [123]. Researchers have observed significant overexpression of SMO, Gli1, and the target gene *FOXM1* in MGs, both aggressive and benign. However, grades II and III MGs exhibit more pronounced changes in the expression of HH pathway genes compared to grade I tumours. Another study found that MGs exhibited HH signalling via IHH and SHH ligands, triggered by SMO [124]. These findings support the development of therapies aimed at inhibiting HH pathway components. Drugs targeting SMO, such as vismodegib and sonidegib, which are already used in other cancers, could be evaluated for efficacy in MGs [123]. Furthermore, emergence of drug resistance necessitates the need for the development of potent inhibitors and combination therapies that can effectively target and overcome resistance mechanisms within the HH pathway [125].

Recent studies have explored the correlation between mesenchymal stem cell markers and tumour grade in greater depth. Researchers focussed on Frizzled 9, GFAP, CD133, Vimentin, and SSEA4, which are associated with stem cell self-renewal, differentiation, and tumour initiation [9]. Similar to HH pathway genes, these markers are significantly higher in grade II/III MGs. Specifically, the Wnt receptor Frizzled 9 is differentially expressed in more aggressive malignant cases [126]. In addition, glial fibrillary acidic protein (GFAP), typically expressed in NSCs, is found at higher levels in grade III MGs, suggesting a correlation between stem cell-like properties and tumour aggressiveness. Three-dimensional spatial analysis has also been used to assess the complex distribution patterns of MgSC markers, revealing that regions with co-expression of multiple markers are associated with higher grade tumours [9]. Another study found CD133-positive cells in 79% of MMGs (WHO grade III). This indicates that CSC niches contribute to tumour progression, emphasising the need for multiple markers to accurately identify CSC subpopulations for effective therapeutic strategies [107].

Despite major advancements in identifying tumour markers in aggressive MGs, a strong link to drug resistance could not be established due to deficiencies in the cell number requirements [127]. Further studies aiming to find this relation, using whole transcriptome microarray analysis have identified differentially expressed stem cell-related pathways between pleomorphic (NG type) and monomorphic (G type) MG cell lines. NG type cell lines, characterised by higher proliferation rates and migration ability, exhibit lower nuclear Caspase-3 expression and higher co-expression of CD133 and Sox2 or AGR2 and BMI1 [85]. These markers are associated with enhanced drug resistance to cisplatin and etoposide, as evidenced by lower levels of nuclear Caspase-3 in treated cells. The identification of these stem cell-associated genes underscores the importance of targeting CSCs to overcome drug resistance in aggressive meningiomas.

### Targeting TME within MGs

In addition to signalling pathways, the TME and CSCs play critical roles in the progression and resistance of MMGs.

Studies have investigated the role of colony-stimulating factor 1 (CSF1) and its receptor (CSF1R) in the TME of MMGs. CSF1 is essential for the survival and differentiation of macrophages, as was shown by Pyonteck et al. (2013), where a cohort of treated mice with glioma survived a median of 5.7 weeks, while 64.3% of those treated with CSF1 blockade were still alive at 26 weeks [128]. Similar roles of CSF1 have been observed in MGs, particularly in the prevalent immunosuppressive M2 phenotype. Using a novel murine model (MGS1) that recapitulates the human MG TME, researchers demonstrated that treatment with anti-CSF1/CSF1R antibodies reprogrammed the primary immunosuppressive myeloid cells within the TME. RNA sequencing (RNA-seq) and mass cytometry revealed that this reprogramming led to a significant reduction in tumour growth without notable effects on T cells. This finding highlights the potential of targeting the CSF1/CSF1R axis as a therapeutic strategy to reduce immunosuppressive myeloid cells [129].

The expression of programmed death-ligand 1 (PD-L1) in MGs has also been explored. PD-L1 is an immune checkpoint protein that binds to the PD-1 receptor on T cells, leading to immune suppression. Tumours often exploit this mechanism to evade immune detection. Studies utilising immunohistochemistry on a cohort of 96 MG cases across grades I to III quantified PD-L1 expression and its correlation with immune cell infiltration and patient outcomes [130]. Results indicated that higher grade MGs exhibited increased PD-L1 expression, particularly in CD68 macrophages, which was associated with worse overall prognosis. This suggests the potential use of immune checkpoint inhibitors, such as PD-1/PD-L1 blockers, as therapeutic agents for these tumours and highlights the need for future clinical trials [130]. In addition, studies have demonstrated an increased presence of immunosuppressive myeloid cells (CD45 + CD11b + PD-L1 +), myeloid-derived suppressor cells (MDSCs), and regulatory T cells (Tregs) in both peripheral blood and tumour tissues of MG patients. These findings are consistent with the current literature, indicating that high-grade MGs have significantly higher levels of PD-L1 expression and increased T-cell infiltration [131].

In attempts to address these elevated PD-L1 levels, PD-1 blockade therapy has been employed. A small clinical trial demonstrated PD-1 blockade neoadjuvant therapy was associated with improved progression-free and overall survival rates [132]. In another study, patients with a mismatch repair (MMR)-deficient MG exhibited a dramatic response to this therapy. MMR deficiency leads to microsatellite instability and the accumulation of mutations, which can generate neoantigens recognised by the immune system, making tumours more susceptible to immunotherapy [133]. Consequently, the patient with MMR-deficient MG showed significant immune activation and clinical response to the treatment, indicating that genetic profiling could help identify patients who would benefit most from immune checkpoint inhibitors [133].

### Targeting tumour cells mitochondria

Given their essential role in cellular homeostasis and tumour growth, mitochondria have emerged as a promising therapeutic target for the treatment of MG CSCs. This recognition has led to a surge of interest in the development of novel pharmacological approaches that specifically target mitochondrial function.

Cancer cells, including CSCs, often exhibit altered metabolism with a significant reliance on mitochondrial OXPHOS for energy production. One of the most extensively studied strategies is to interfere with OXPHOS by inhibiting the electron transport chain (ETC) [134]. Diabetic drugs such as metformin have shown efficacy in this regard, prompting further research into other compounds [135]. Antibiotics such as antimycin A, oligomycin, and monoamine oxidase B (MAO-B) inhibitors used in Parkinson’s disease, and reactive oxygen species (ROS) inducers such as menadione have also been investigated for their potential to disrupt mitochondrial metabolism and reduce tumour viability [136–138]. Another approach focuses on inducing mitochondrial dysfunction by disrupting mitochondrial dynamics to preserve mitochondrial integrity and function [134]. Inhibitors of mitochondrial fission, such as Mdivi-1, have demonstrated efficacy in reducing CSC viability by reducing proliferation and inducing apoptosis in tumour cells [139].

A recent promising strategy is to target the apoptotic pathways of the mitochondria. CSCs often escape apoptosis by overexpressing anti-apoptotic proteins such as Bcl-2, which are located on the mitochondrial outer membrane. By inhibiting these proteins with BH3 mimetics, the apoptotic capacity of CSCs can be restored, ultimately leading to their destruction [140]. In addition, inhibition of mitochondrial protein translation has emerged as a novel therapeutic strategy in cancer treatment, with the well-known antibiotic tigecycline showing particular promise [141].

### Advancing from traditional therapies: the effectiveness of combination therapies

Traditional therapies, including surgery and radiation, often fall short in treating MMG, necessitating the exploration of more effective treatment strategies. One promising approach is targeting MG CSCs through combination therapies.

Immune checkpoint inhibitors have shown effectiveness, yet finding a universal drug effective across multiple tumour types would be even more advantageous. To initiate this process, the TME of two tumour types, MGs and GBs, were assessed for similarities in their immunosuppressive elements. Both exhibited high levels of regulatory T cells (Tregs) and TAMs, as well as increased cytokines promoting Treg differentiation and the presence of indoleamine 2,3-dioxygenase 1 (IDO1), which further contributes to immunosuppression. These findings suggest that both tumour types share a similar immunosuppressive microenvironment, which could be targeted by immunomodulatory therapies [142]. Combining immune checkpoint inhibitors with other immunomodulatory treatments, such as IDO1 inhibitors, could be an effective strategy for treating multiple tumour types.

Hydroxyurea (HU) has been used in treating MGs due to its ability to inhibit ribonucleotide reductase, hindering DNA synthesis [78]. However, its effectiveness as a monotherapy is limited, necessitating combination with drugs providing synergistic effects. Calcium channel antagonists, which exploit the overexpression of voltage-gated calcium channels in cancer cells crucial for tumour survival, show promise [143]. Combining HU with non-specific calcium channel antagonist diltiazem or high voltage-activated calcium channel antagonist verapamil significantly decreased tumour size and cell number in MG cell lines compared to either agent alone [144].

*NF2* deficiency is a common genetic alteration in MMGs. The loss of the *NF2* gene results in the activation of multiple oncogenic pathways. Researchers have investigated the effectiveness of mTORC1/2 inhibitors with dasatinib, a tyrosine kinase inhibitor targeting EPH receptor tyrosine kinases, in *NF2*-null MG cells. This combination led to substantial inhibition of tumour growth with no crosstalk with mTORC1/2 signalling and minimal adaptive changes post-treatment [145, 146].

Radiation therapy remains the gold standard for treating unresectable MGs. However, its inconsistent effectiveness in malignant cases has driven research towards identifying small molecule inhibitors that can enhance its efficacy. Among these, inhibitors targeting the VEGF pathway have shown promise in synergising with radiotherapy [145]. The potential mechanisms by which angiogenesis inhibitors may enhance radiosensitivity include direct antitumor effects, endothelial cell radiosensitisation leading to damaged tumour vasculature, and improved oxygenation resulting from the elimination of ineffective tumour vessels and decreased interstitial pressure [145].

Innovative combination therapies continue to emerge, aiming to target multiple molecular pathways simultaneously. Researchers have discussed the potential of combining everolimus with octreotide, bevacizumab, and sunitinib for treating recurrent MGs. These combinations target various pathways involved in tumour growth and angiogenesis, offering a multi-faceted approach to treatment [147]. Everolimus and octreotide target the mTOR pathway and somatostatin receptors, respectively. Combining these with angiogenesis inhibitors such as bevacizumab or sunitinib, which target multiple tyrosine kinases, shows high activity in treating recurrent MGs [147]. Clinical studies suggest these combinations are promising, and future research is focussing on a wider variety of drug combinations, including peptide receptor radionuclide therapy (PRRT) using radiolabelled somatostatin analogs to target somatostatin receptors on MG cells [148].

Valproic acid (VPA), a commonly used anti-epileptic drug, has shown promise in enhancing radiosensitivity of MG stem-like cells. VPA has been proven to reduce the growth of both MG sphere cells and MG adherent cells (MgACs). Studies have revealed that VPA treatment increases the expression of phosphorylated cdc2 (p-cdc2) and phosphorylated H2AX (p-H2AX), crucial markers of DNA damage response [149]. Furthermore, VPA treatment upregulated cleaved caspase-3 and PARP, initiating the activation of apoptotic pathways in MgSCs. Importantly, the combined treatment with VPA and irradiation further decreased the expression of Oct-4, a stem cell marker, suggesting that VPA enhances radiosensitivity by targeting the stem-like properties of MgSCs [149].

### Gene editing technologies—using CRISPR/Cas9 to prevent MG formation

Recent advancements in gene-editing technologies, particularly CRISPR/Cas9, have opened new avenues for understanding MMGs and developing targeted therapeutic strategies.

The *NF2* gene is frequently mutated in MGs, and its product, merlin, acts as a tumour suppressor by regulating cell growth and proliferation [150]. Researchers have employed CRISPR/Cas9 to create MG cell models within *NF2* knockout mice. This knockout resulted in the absence of merlin, leading to significant changes at both the cellular and molecular levels [151]. Merlin interacts with several signalling pathways, including the Hippo pathway, which regulates cell proliferation and apoptosis [152]. *NF2*-depleted MG cells exhibited reduced apoptosis and increased colony formation, indicating enhanced proliferative capacity. These cells also showed increased activity in pathways associated with cell survival and proliferation, such as the PI3K/AKT pathway [151]. Table 3 summarises the therapeutic strategies and its effects on MgSCs.

**Table 3:** Current therapeutic strategies targeting meningioma stem cells

Therapeutic strategies	Effect on MgSCs
Therapies targeting MgSC markers and their related signalling pathways	
HH pathway inhibition (e.g. vismodegib and sonidegib) [123–125]	Overexpression of SMO, Gli1, and FOXM1 in MGs; more pronounced in higher grades; potential for inhibiting HH pathway components
Targeting Wnt receptor Frizzled 9, GFAP, CD133, Vimentin, SSEA4 [9, 107, 126]	Higher expression in aggressive MGs; correlation with tumour grade and aggressiveness
Targeting TME within MGs	
CSF1/CSF1R inhibition [129]	Reprogramming immunosuppressive myeloid cells, reducing tumour growth
PD-1/PD-L1 blockade [130, 132, 133]	Increased PD-L1 expression in high-grade MGs; potential for immune checkpoint inhibitors
Targeting tumour cells mitochondria	
OXPHOS blockage using ETC inhibition (metformin, antimycin A, oligomycin, MAO-B inhibitors, and menadione) [134–138]	Disrupting mitochondrial metabolism and reducing tumour viability
Mitochondrial fission inhibitors (Mdivi-1) [134, 139]	Disruption of mitochondrial dynamics; reducing proliferation and inducing apoptosis in tumour cells
Anti-apoptotic protein inhibitors (BH3 mimetics) [140]	Restoring apoptotic capacity of MgSCs
Antibiotic therapy (tigecycline) [141]	Inhibition of mitochondrial protein translation
Combination therapies	
HU with calcium channel antagonists [78, 143, 144]	Synergistic effects reducing tumour size and cell number
mTORC1/2 inhibitors with dasatinib [145, 146]	Effective in NF2-null MG cells
Radiation therapy with small molecule inhibitors [145]	Targeting VEGF pathways for more effective treatment
Everolimus with octreotide, bevacizumab, sunitinib [147]	Multi-faceted approach targeting tumour growth and angiogenesis
VPA with irradiation [149]	Enhancing radiosensitivity and reducing stem-like properties of MgSCs
Gene-editing technologies	
CRISPR/Cas9-targeting NF2 gene [150–152]	Creating meningioma models, revealing pathways related to cell growth and proliferation

*HH*: hedgehog, *MgSC*: meningioma stem cell, *Wnt*: wingless-related integration site, *GFAP*: glial fibrillary acidic protein, *SSEA 4*: stage-specific embryonic antigen 4, *CSF1*: colony-stimulating factor 1, *CSF1R*: colony-stimulating factor 1 receptor, *TME*: tumour microenvironment, *PD-1*: programmed cell death 1, *PD-L1*: programmed death-ligand 1, *mTORC1*: mammalian target of rapamycin complex 1, *CRISPR*: clustered regularly interspaced short palindromic repeats, *Cas9*: CRISPR-associated protein 9, *SMO*: smoothened, *GLI1*: glioma-associated oncogene homolog 1, *FOXM1*: forkhead box protein M1, *NF2*: neurofibromatosis type 2, *VEGF*: vascular endothelial growth factor, *DNA*: deoxyribonucleic acid, *VPA*: valproic acid, ETC: electron transport chain

## Limitations in meningioma research

One of the most prominent limitations in MG research is the small sample size. Most studies rely on cell lines and animal models, which may fully represent the human condition. While useful for preliminary findings, these models lack the genetic diversity of human populations, complicating the assessment of therapy efficacy across diverse populations [144]. In addition, the reliance on animal models makes it challenging to evaluate the long-term effectiveness and safety of combination therapies. The focus on short-term outcomes, such as tumour size reduction and cell proliferation rates, often overlooks the potential for recurrence, complicating conclusions about long-term side effects and sustained effectiveness [146].

MGs exhibit significant genetic, epigenetic, and phenotypic heterogeneity, making the development of universal treatment protocols difficult. Many studies do not account for this variability, often reviewing targeted agents without addressing specific MG subtypes [145]. The few studies that do consider subtypes are typically in preclinical or early clinical trial stages. While researchers emphasise the potential of targeting multiple molecular pathways, these conclusions are primarily based on genetic and epigenetic analyses. Clinical trials are essential to validate these findings in human patients [153].

Ethical constraints also limit the scope of research, particularly in clinical trials. The aggressive nature of high-grade MGs means that patients may not have the time to participate in lengthy trials. Furthermore, some patient cohorts may not be able to participate in trials, such as paediatric patients, pregnant women, and individuals with multiple comorbidities, due to the vulnerable state these populations are already in Ref. [154]. In addition, the ethical implications of *CRISPR* gene editing, especially germline modifications, are significant. Germline changes are heritable, raising concerns about unintended long-term effects and potential new genetic disorders in future generations, complicating the translation of CRISPR-based findings to clinical settings [151].

CRISPR/Cas9 technology, despite its precision, is not without flaws. Off-target effects, where CRISPR inadvertently edits unintended parts of the genome, pose significant concerns. Although methods exist to minimise these effects, they cannot be entirely eliminated. The efficiency of CRISPR in targeting specific genes varies, leading to inconsistent results across different studies. Further research is necessary to validate CRISPR/Cas9 screen results due to potential off-target effects and variable efficiency in different cell lines. Moreover, while CRISPR technology can effectively disrupt specific genes, its therapeutic efficacy in real-world scenarios may be limited by tumours’ adaptive resistance mechanisms [155].

## Conclusion

In conclusion, exploring MgSC-specific molecular mechanisms and markers is crucial for identifying novel targets for early intervention and prevention strategies. A thorough understanding of MgSCs’ roles in therapy resistance, tumour recurrence, and metastasis in MMGs is essential for overcoming resistance to conventional treatments. Targeting MgSCs through therapies such as inhibitors of key signalling pathways (e.g. Wnt, Notch, and HH) could address tumour persistence and lead to more durable regression. Advancements in MG treatment will necessitate a multidisciplinary approach that integrates molecular biology, genetics, and clinical research. For instance, the *CRISPR/Cas9* gene editing highlights the importance of collaboration between clinicians and researchers to ensure the therapy’s safety and efficacy. Optimising personalised medicine by tailoring treatments to specific CSCs holds considerable promise. By leveraging genetic and molecular profiling of individual tumours, personalised medicine can pinpoint CSC markers and pathways unique to each patient. Future research should prioritise identifying novel biomarkers for early detection and monitoring treatment response. In addition, combining targeted therapies with immunotherapy and exploring nanotechnology for precise drug delivery present promising avenues for advancing treatment strategies.

## Data Availability

Not applicable.

## Abbreviations

CNS: Central nervous system
MMGs: Malignant meningiomas
MGs: Meningiomas
MRI: Magnetic resonance imaging
CSCs: Cancer stem cells
NF2: Neurofibromatosis 2
KLF4: Krüppel-like factor 4
MEN1: Multiple endocrine neoplasia type 1
AML: Acute myeloid leukaemia
HATs: Histone acetylases
TRAF7: Tumour necrosis receptor-associated factor
ABC: Adenosine-triphosphate-binding cassette
TME: Tumour microenvironment
MgSCs: Meningioma stem cells
HH: Hedgehog
SHH: Sonic hedgehog
IHH: Indian hedgehog
PTCH1: Patched receptor 1
GFAP: Glial fibrillary acidic protein
CSF1: Colony-stimulating factor 1
RNA-seq: Ribonucleic acid sequencing
PD-L1: Programmed death-ligand 1
MDSCs: Myeloid-derived suppressor cells
Tregs: Regulatory T cells
MMR: Mismatch repair
IDO1: Indoleamine 2,3-dioxygenase 1
HU: Hydroxyurea
PRRT: Peptide receptor radionuclide therapy
VPA: Valproic acid
MgACs: Meningioma-adherent cells
P-cdc2: Phosphorylated cdc2
EMBASE: Excerpta Medica Database
MEDLINE: Medical literature analysis and retrieval system online
CRISPR: Clustered regularly interspaced short palindromic repeats
WHO: World Health Organisation
CBTRUS: Central Brain Tumour Registry of the United States
DNA: Deoxyribonucleic acid
DNMT: Deoxyribonucleic acid methyltransferase
VEGF: Vascular endothelial growth factor
ECM: Extracellular matrix
TNF: Tumour necrosis factor
NSC: Neural stem cell
ESCs: Embryonic stem cells
Oct-4: Octamer binding transcription factor 4
Sox2: Sex determining region Y (SRY)-box 2
Nestin: Neuroepithelial stem cell protein
SMO: Smoothened
Gli1: Glioma-associated oncogene homolog
CRISPR: Clustered regularly interspaced short palindromic repeats
Cas9: CRISPR-associated protein 9
mTOR: Mammalian target of rapamycin
GB: Glioblastoma
